# Effects of Different Ligands in the Notch Signaling Pathway on the Proliferation and Transdifferentiation of Primary Type II Alveolar Epithelial Cells

**DOI:** 10.3389/fped.2020.00452

**Published:** 2020-08-06

**Authors:** Xiuxiang Liu, Xiaoxi Zhu, Guoqing Zhu, Chaoyun Wang, Ruiwei Gao, Jinshuai Ma

**Affiliations:** ^1^Department of Neonatology, Binzhou Medical University Hospital, Binzhou, China; ^2^Department of Neonatology, Yantai Affiliated Hospital of Binzhou Medical University, Yantai, China; ^3^Department of Pediatrics, Binzhou People's Hospital, Binzhou, China; ^4^School of Pharmaceutical Sciences, Binzhou Medical University, Yantai, China; ^5^Department of Neonatology, Children's Hospital of Fudan University, Shanghai, China; ^6^Department of Neonatology, Binzhou Medical University Hospital, Binzhou, China

**Keywords:** alveolar type II epithelial cells, Notch, Dlk1, Jagged1, respiratory distress syndrome of newborn

## Abstract

**Background:** Transdifferentiation of type II alveolar epithelial cells (AECII) into type I alveolar epithelial cells (AECI) is involved in neonatal respiratory distress syndrome (NRDS). Different ligands of the Notch pathway could have different effects on AECII transdifferentiation.

**Objective:** To investigate the effects of Dlk1 and Jagged1 on the proliferation and transdifferentiation of AECII.

**Methods:** Fetal AECIIs (19 days of gestation) were divided: control group, Dlk1 group, rhNF-κB group. Proliferation was tested using the MTT assay. Expression of surfactant protein C (SP-C) and aquaporin 5 (AQP5) was examined by immunofluorescence. mRNA and protein levels of SP-C, AQP5, Nortch1, Dlk1, Jagged1, and Hes1 were examined by RT-PCR and western blot.

**Results:** In response to Dlk1, cell number and proliferation were increased (*P* < 0.05), and mRNA and protein levels of SP-C, Dlk1, Notch1, and Hes1 were up-regulated, while AQP and Jagged1 were decreased. In response to rhNF-κB, the cell number and proliferation were reduced, and mRNA and protein levels of Jagged1 and Notch1 were up-regulated, while Dlk1, and SP-C were downregulated. In the Dlk1 group, SP-C, and AQP5 expression patterns suggested that the cells were still transdifferentiating by 96 h, while in the rhNF-κB group, most cells had transdifferentiated by 72 h and were close to apoptosis by 96 h.

**Conclusion:** These results suggest that Dlk1 promoted proliferation of AECIIs and inhibited cell transdifferentiation, while Jagged1 treatment inhibited proliferation of AECIIs and promoted transdifferentiation to AECIs. These results provide some clue for the eventual management of NDRS.

## Introduction

Neonatal respiratory distress syndrome (NRDS) mostly happen in premature infants <37 weeks of gestation. NRDS is characterized by progressive dyspnea, cyanosis, and respiratory failure (1). The main mechanism is lack of pulmonary surfactant, which lead to a serious lack of oxygen. The alveolar epithelium is composed of type I and type II alveolar epithelial cells, and represents 99% of the surface area of the lung (2). It is well-established that alveolar epithelial type II cells (AECII) serve as defenders of the alveoli; their functions include synthesis and secretion of pulmonary surfactant, activation of ion transport, and maintenance and repair of alveolar epithelium after lung injury (3). Primary AECIIs gradually transdifferentiate to type I alveolar epithelial cells (AECI) and then lose the ability to secrete pneumonocyte surfactant (PS) (2). Therefore, if we could regulate and control the process of cell transdifferentiation from AECII to AECI during fetal lung maturation and hence promote the secretion of PS, maybe it could be possible to prevent and treat NRDS (4).

Notch signaling is essential for the development of a variety of tissue and organ through interactions between neighboring cells. It is involved in the precise control of each lineage cell growth, proliferation, differentiation, and cell fate determination (4). Importantly, Notch signaling is involved in lung development and dysregulation of Notch is known to be involved in lung diseases (5). Dlk1 is a non-canonical Notch ligand that plays a variety of roles in cell proliferation and differentiation (6–8). In a previous study by our group, high-throughput sequencing of AECII transdifferentiation revealed the differential expression of a number of genes, including decreased Dlk1, while Jagged1, another ligand involved in the Notch pathway (9), was increased (10). It is well-known that the expression of the different Notch ligands is tightly regulated during fetal lung development (5, 11, 12). RhNF-κB is an activator of the Notch signaling pathways; it cannot activate the Notch receptor, but it can promote the expression of Jagged1 and activate the Notch pathway indirectly (13).

It could be hypothesized that different ligands of the Notch pathway produce different biological effects and have different impacts on cell transdifferentiation and proliferation. Therefore, the aim of the present study was to investigate the effects of Dlk1 and Jagged1 on the proliferation and transdifferentiation of AECII. The results could provide clues about the modulation of AECII transdifferentiation, which could eventually be used to prevent or treat NRDS.

## Materials and Methods

### Animals

Adult Sprague-Dawley rats were bought from the Beijing Vital River Laboratory Animal Technology Co., Ltd. (Beijing, China). The experiments were approved by the Binzhou Medical University Animal Ethics Committee. The procedures followed were in accordance with the standards set forth in the eighth edition of “Guide for the Care and Use of Laboratory Animals.” Female rats (weighing 200–220 g) and male rats (weighing 250–300 g) were housed in the same cage at a female:male ratio of 2:1. The vaginal smears were checked the next morning at 8 a.m. to observe the presence of sperm, which indicated positive mating. This day was considered day zero of pregnancy.

### AECII Isolation and Culture

Pregnant rats on day 19 of gestation were anesthetized and the pups were delivered by cesarean section. Fetal rat lung tissues were quickly removed and submerged in ice-cold phosphate-buffered saline (PBS). Trachea, bronchi, and vascular tissues were cleared off. After washing in PBS, lung tissues were cut into 1-mm^3^ pieces, which were placed in a 15 ml centrifuge tube and washed repeatedly with PBS until the supernatant fluid was clear. Then, lung tissues were fully digested with trypsin (Sigma, St Louis, MO, USA), and type IV collagenase (Sigma, St Louis, MO, USA), filtered through 100-μm mesh sieve, and centrifuged at 1,200 rpm for 8 min. The supernatant was discarded. The digested tissue pellet was resuspended in 20 ml of DMEM medium containing 12% FBS (Hyclone, Thermo Fisher Scientific, Waltham, MA, USA) and plated in rat IgG-coated tissue culture flasks. The medium containing non-adherent cells was aspirated 40 min later and transferred into another rat IgG-coated tissue culture flask. After repeating this step three times, the non-adherent cells consisting of AECII were finally collected from the flask (6).

The AECII were centrifuged, resuspended, adjusted to 2–5 × 10^5^/ml, plated on a culture dish, and incubated in a 37°C CO_2_ incubator. After 24 h, the cells were divided into three groups: (1) the control group, cultured with DMEM, and 12% FBS; (2) the Dlk1 group: cells were cultured with Dlk1 (Abnova, Taipei, Taiwan) at a final concentration of 100 ng/mL; and (3) the rhNF-κB group: cells were cultured with rhNF-κB (Promega, Madison, WI, USA) at a final concentration of 1 gsu/ml. The culture media were changed every 24 h.

### Assessment of Cell Number

The filtered and purified AECIIs were suspended and adjusted to a cell density of ~4 × 105/ml. The cell suspension was plated in 24-well plates, 0.5 ml/well, and kept in a 5% CO_2_ incubator at 37°C. Cells were collected and digested with 0.25% trypsin every 24 h, and then cell numbers were counted under a microscope (Olympus Corporation, Tokyo, Japan), The mean number of cells in the five wells was considered the cell number for each sample.

### Cell Proliferation Assay

Cell viability was determined using Methyl thiazolyl tetrazolium (MTT) at each time point. The AECIIs were inoculated in 96-well plates at 6 × 104/ml, 200 μl/well. The medium was removed after 24, 48, 72, and 96 h. The cells were washed twice with PBS, and 20 μl of MTT (Sigma, Aldrich) per well was added to wells and incubated for an additional 4 h. The supernatant was removed carefully, 150 μl of DMSO (Sigma, St Louis, MO, USA) were added, the plates were mixed for 10 min, and the absorbance was measured in each well at 570 nm in a microplate reader (Thermo, Waltham, MA). Fice wells were measured for each group and the mean value was used for analysis.

### Electron Microscopy

Electron microscopy was used to observe the ultrastructure of cells at each time point. Cells were collected at 48, 72, and 96 h, digested with 0.25% trypsin, centrifuged at 2,000 rpm for 5 min, and fixed with 3% glutaraldehyde in cacodylate buffer (pH 7.2) for 30 min. Then, the samples were post-fixed in 1% osmium tetroxide in 0.1 M cacodylate buffer, transferred to 50% ethanol, and centrifuged. The resultant samples were encased in molten agar. The agar blocks were dehydrated, infiltrated with araldite epoxy resin, and embedded in molds. The resin was polymerized at 60°C for 24 h. Semi-thin (1 μm) and ultrathin (60–80 nm) sections were cut using a Reichert-Jung Ultracut E ultramicrotome. For selection of areas of interest on light microscopy, the semithin sections were stained using 0.8% toluidine blue in 0.8% borax containing 0.16% pyronin Y. The ultrathin sections were stained sequentially with a saturated solution of uranyl acetate in 50% ethanol and Reynold's lead citrate. Ultrathin sections were observed with a CM10 transmission electron microscope (Philips, Best, The Netherlands).

### Immunofluorescence

The expression of SP-C and AQP5 was observed with confocal laser scanning microscopy (C1-SHS, Nikon, Tokyo, Japan) at each time point. AECIIs were kept in laser confocal special dishes. Cells in these dishes were fixed with 4% paraformaldehyde for 10 min and washed three times with PBS. The primary antibodies sheep anti-SP-C and rabbit anti-AQP5 (Santa Cruz Biotechnology, Santa Cruz, CA, USA) were added and the dishes were kept in a wet box at 4°C for 24 h. The dishes were washed three times with PBS and anti-sheep IgG conjugated with FITC or anti-rabbit IgG conjugated with Texas Red (Sigma, St Louis, MO, USA) was added and incubated at 37°C for 2 h, followed by washing three times with PBS. One milliliter of 10 ng/mL Hoechst 33,342 (Sigma, St Louis, MO, USA) was added and incubated at room temperature for 20 min. After washing with PBS, cells were mounted in Vectashield mounting medium. Finally, cells were observed with a C1-SHS laser confocal microscope (Nikon, Tokyo, Japan).

### Detection of mRNA Expression of SP-C, AQP5, Dlk1, Jagged1, Notch1, and Hes1

SP-C, AQP5, Dlk1, Jagged1, Notch1, and Hes1 mRNAs were detected by reverse transcription (RT)-PCR (ABI 9700, Applied Biosystems, Foster City, CA, USA). The amplified PCR products were resolved in agarose and visualized at 260 nm. The amount of mRNA was calculated relative to β-actin. Total RNA was extracted from each group using TRIzol (Invitrogen Inc., Carlsbad, CA, USA). RNA purity was estimated by calculating the 260/280 nm absorbance and reverse transcribed into cDNA using a RT-PCR kit (Fermentas, Thermo Fisher Scientific, Waltham, MA, USA), according to the manufacturers' instructions. Relative levels of target gene mRNA expression were determined by the PCR system (Corbett iQ5 Opital Module, Corbett, USA) using the cDNA as a template and specific primers synthesized by Sangon Biotech (Shanghai, China) (Table 1). PCR reactions were performed in duplicate at 95°C for 5 min and subjected to 40 cycles of 95°C for 15 s, 56–59°C for 30 s, and 72°C for 30 s. The cycle threshold (CT) of each sample were analyzed by the Sequence Detection System software (version 110; Applied Biosystems, Foster City, CA, USA).

**Table 1:** Primer sequences for RT-PCR.

	**Forward (5**′**->3**′**)**	**Reverse (5**′**->3**′**)**
SP-C	CTGAGATGGTCCTTGAGATGAG	GTAGCGATGGTGTCTGTGTGTT
AQP5	CTCCCAACCCAGTATCTCAAGT	GCCATCTATCCCTCTCCTGAAG
Dlk1	ACAAGGAGGCTGGTGATGAG	GTGAGGAACCCCGATAATGTAG
Jagged1	ATTCGATCTACATAGCCTGTGAG	CTATACGATGTATTCCATCCGGT
Notch1	CAGCGAATCCGAGGACTATG	CAGGCGTGTTGTTCTCACAG
HES1	GCAGCAGCAGTTGCAAGCTC	TCTTGTCATCTTTTATTGCC
B-actin	CACCCGCGAGTACAACCTTC	CCCATACCCACCATCACACC

### Western Blot

The cells were harvested and homogenized in ice-cold homogenization buffer (10 mM Tris-HCl, pH 7.4, 1.5 mM EDTA, pH 8.0, and 100 mg/l phenylmethylsulfonyl fluoride; Sigma, St Louis, MO, USA), followed by centrifugation at 20,000 g for 20 min at 4°C. Supernatants were collected and protein concentrations were determined using Coomassie brilliant blue. Total proteins (100 μg/lane) were separated by 10% SDS-PAGE and transferred onto polyvinylidene fluoride (PVDF) membranes (Millipore Corp., Billerica, MA, USA). After being blocked with 5% non-fat dry milk in Tris-buffered saline/Tween-20 (TBST) buffer for 2 h, the membranes were incubated overnight at 4°C with rabbit polyclonal antibodies against SP-C (1:1,000; Biosynthesis Biotechnology Co., Ltd., Beijing, China), AQP5 (1:1,000; Biosynthesis Biotechnology Co., Ltd., Beijing, China), and β-actin (1:1,000; Biosynthesis Biotechnology Co., Ltd., Beijing, China), rabbit polyclonal antibodies against Dlk1 (1:500; Santa Cruz Biotechnology, Santa Cruz, CA, USA), Notch1 (1:500; Santa Cruz Biotechnology, Santa Cruz, CA, USA), Jagged1 (1:500; Santa Cruz Biotechnology, Santa Cruz, CA, USA), and Hes1 (1:500; Santa Cruz Biotechnology, Santa Cruz, CA, USA). Subsequently, the membranes were incubated with horseradish peroxidase-conjugated goat anti-rabbit IgG (1:5,000; Amersham, GE Healthcare, Waukesha, WI, USA) at 37°C for 2 h. After TBST washing, enhanced chemiluminescence (ECL) reagent (Tiangen Biotech Co., Ltd., Beijing, China) was added to the membranes. Western blot bands were read for integrated optical density (IOD) and quantified using LabWorks 4.5 software (UVP Inc., Upland, CA, USA).

### Statistical Analysis

All data were presented as mean ± standard deviation (SD) and analyses were performed using SPSS 17.0 (IBM, Armonk, NY, USA). The interaction effect of incubation time and culture group was analyzed by two-way ANOVA. The Bonferroni method was used to correct for multiple comparisons. Two-sided *P* < 0.05 were considered statistically significant.

## Results

### Effect of Dlk1 and rhNF-κB on the Active of Notch Pathway

**Figure 1:**
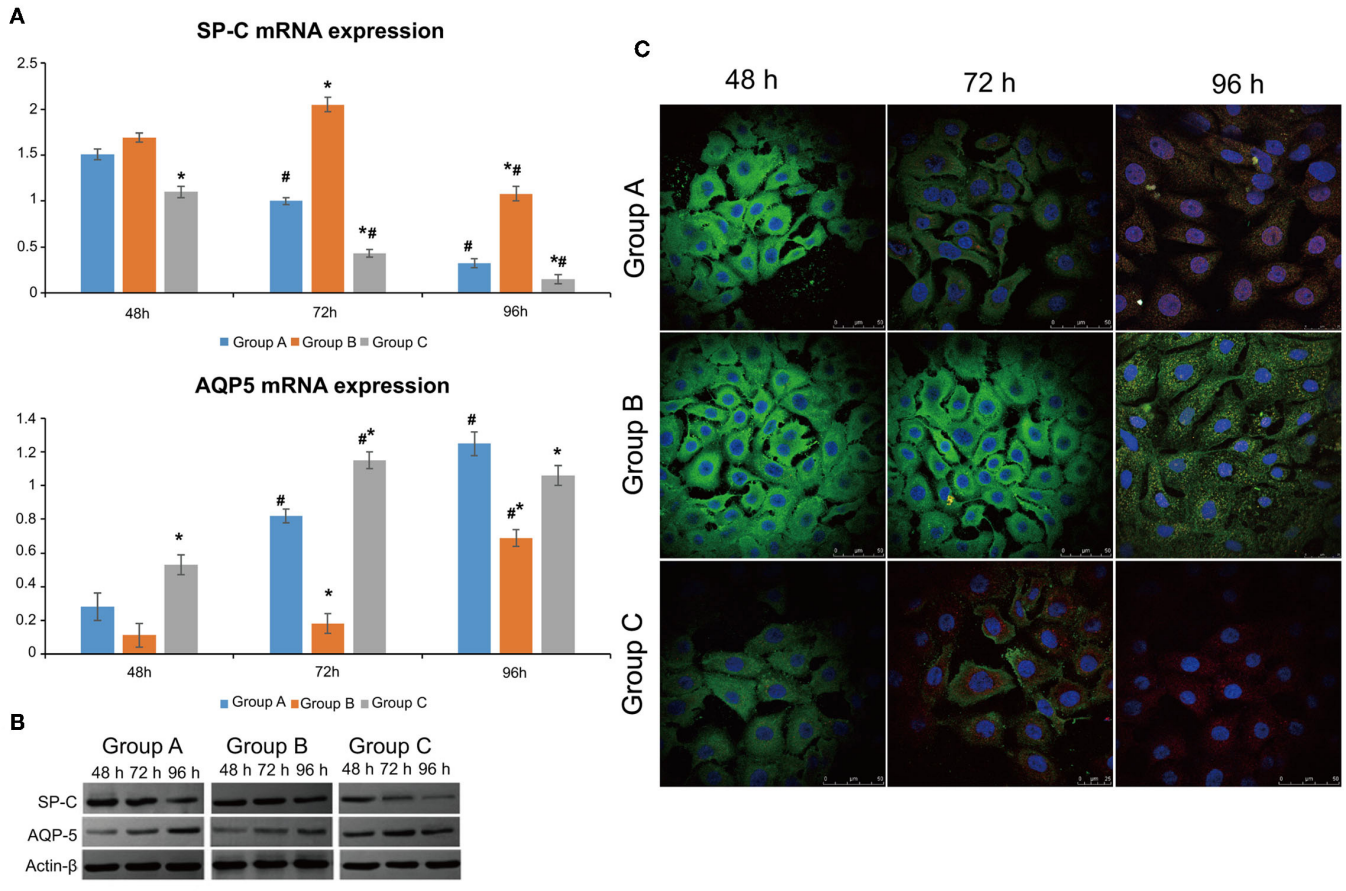
Effect of Dlk1 and rhNF-κB on the transdifferentiation of primary cultured rat type II alveolar epithelial cells (AECII). AECIIs were divided into three groups: (1) the control group, cultured with DMEM, and 12% FBS; (2) the Dlk1 group: cells were cultured with Dlk1 (Abnova, Taipei, Taiwan) at a final concentration of 100 ng/mL; and (3) the rhNF-κB group: cells were cultured with rhNF-κB (Promega, Madison, WI, USA) at a final concentration of 1 gsu/ml. **(A)** After 24, 48, and 72 h of treatment, SP-C and AQP5 mRNA expression levels were determined by RT-PCR. *P < 0.05 vs. the control group at the same time point; ^#^*P* < 0.05 vs. 24 h of the same group. **(B)** After 48, 72, and 96 h of treatment, SP-C and AQP5 protein expression levels were determined by western blot. ß-actin was used as control. Loading controls were reused in Figure 2. *P < 0.05 vs. the control group at the same time point; ^#^*P* < 0.05 vs. 48 h of the same group. Data are shown as mean ± standard deviation (SD) of 3 independent experiments each conducted in 5. **(C)** SP-C and AQP5 protein locations were determined by immunofluorescence. Group A = control group; Group B = Dlk1 group; Group C = rhNF-κB group.

SP-C mRNA expression declined with time in the control and rhNF-κB groups (*P* < 0.05), while it peaked at 48 h (*P* > 0.05) and then decreased in the Dlk1 group (*P* < 0.05). SP-C mRNA expression was higher in the Dlk1 group compared with controls (*P* < 0.05), while it was lower in the rhNF-κB group (Figure 1A). AQP5 mRNA expression was lower in the Dlk1 and rhNF-κB groups compared with controls (*P* < 0.05; Figure 1A). The protein expression levels of Sp-C and AQP5 followed similar patterns (Figure 1B).

**Figure 2:**
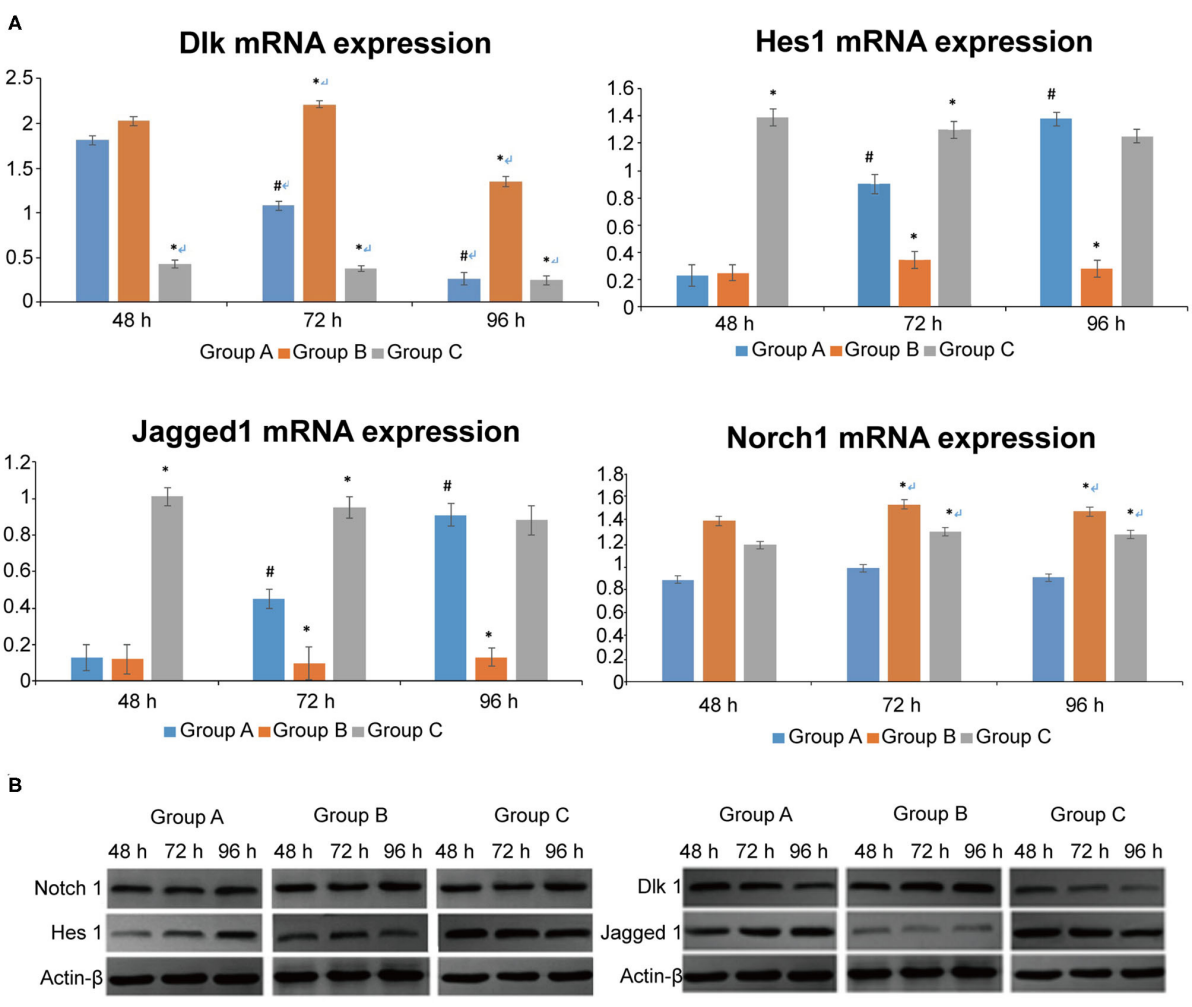
Effect of Dlk1 and rhNF-κB on the Notch signaling pathway in primary cultured rat type II alveolar epithelial cells (AECII). AECIIs were divided into three groups: (1) the control group, cultured with DMEM, and 12% FBS; (2) the Dlk1 group: cells were cultured with Dlk1 (Abnova, Taipei, Taiwan) at a final concentration of 100 ng/mL; and (3) the rhNF-κB group: cells were cultured with rhNF-κB (Promega, Madison, WI, USA) at a final concentration of 1 gsu/ml. **(A)** After 24, 48, and 72 h of treatment, Dlk1, Jagged1, Nortch1, and Hes1 mRNA expression levels were determined by RT-PCR. *P < 0.05 vs. the control group at the same time point; ^#^*P* < 0.05 vs. 48 h of the same group. **(B)** After 48, 72, and 96 h of treatment, Dlk1, Jagged1, Nortch1, and Hes1 protein expression levels were determined by western blot. ß-actin was used as control. Loading controls were reused in Figure 1. *P < 0.05 vs. control group at the same time point; ^#^*P* < 0.05 vs. 48 h of the same group. Data are shown as mean ± standard deviation (SD) of 3 independent experiments each conducted in 5. Group A = control group; Group B = Dlk1 group; Group C = rhNF-κB group.

Dlk1 expression decreased in time in the control group (*P* < 0.05), but did not change in the Dlk1 and rhNF-κB groups (Figure 2A). Dlk1 mRNA expression was higher in the Dlk1 group compared with controls at all-time points (*P* < 0.05), while it was lower than controls in the rhNF-κB group at all-time points (*P* < 0.05) (Figure 2A). Hes1 expression increased in time in the control group (*P* < 0.05), but did not change in the Dlk1 and rhNF-κB groups (Figure 2A). Hes1 mRNA expression was lower in the Dlk1 group compared with controls at 48 and 96 h (*P* < 0.05), while it was higher than controls in the rhNF-κB group at 24 and 48 h (*P* < 0.05; Figure 2A). Jagged1 expression increased in time in the control group (*P* < 0.05), but did not change in the Dlk1 and rhNF-κB groups (Figure 2A). Jagged1 mRNA expression was lower in the Dlk1 group compared with controls at 48 and 96 h (*P* < 0.05), while it was higher than controls in the rhNF-κB group at 24 and 48 h (*P* < 0.05; Figure 2A). Notch1 mRNA expression was higher in the Dlk1 and in the rhNF-κB groups compared with controls at 48 and 96 h (*P* < 0.05; Figure 2A). Protein expression levels followed similar patterns (Figure 2B).

These results suggest that both Dlk1 and Jagged1 can activate the Notch pathway, but had opposite effects on AECII transdifferentiation.

### Dlk1 Increased AECII Proliferation, While rhNF-κB Decreased Their Proliferation

**Figure 3:**
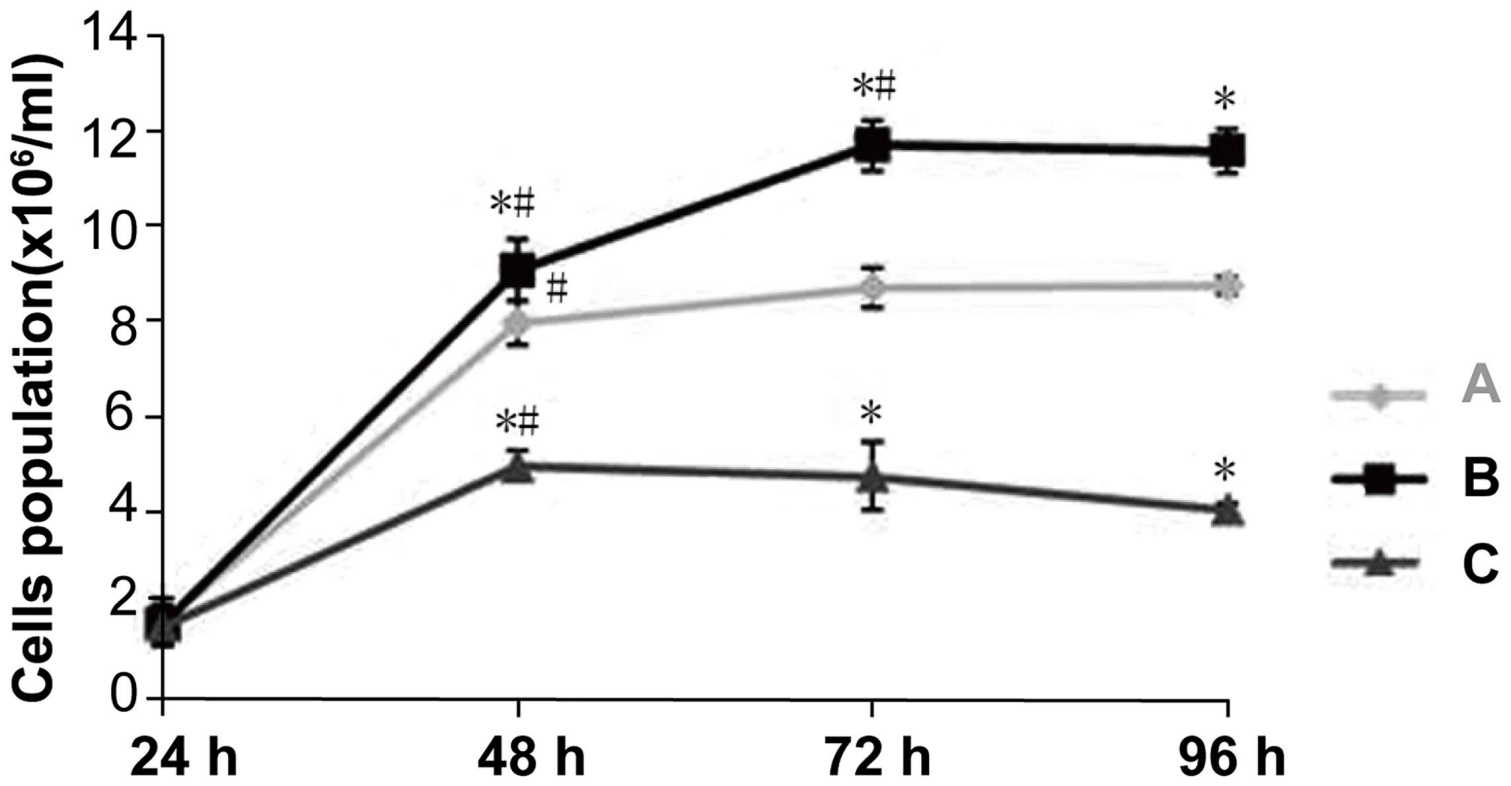
Effect of Dlk1 and rhNF-κB on the proliferation of primary cultured rat type II alveolar epithelial cells (AECII). AECIIs were divided into three groups: (1) the control group, cultured with DMEM, and 12% FBS; (2) the Dlk1 group: cells were cultured with Dlk1 (Abnova, Taipei, Taiwan) at a final concentration of 100 ng/mL; and (3) the rhNF-κB group: cells were cultured with rhNF-κB (Promega, Madison, WI, USA) at a final concentration of 1 gsu/ml. Cells were incubated for 24, 48, 72, and 96 h. (A) Cell numbers were determined by cell counting plates under light microscopy. Cell growth curves were made. (Table 2) Cell Proliferation was determined by MTT. Cell proliferation curves are shown as absorption value. Data are shown as mean ± SD of 3 independent experiments each conducted in 5. *P < 0.05 vs. the control group at the same time point; ^#^*P* < 0.05 vs. 24 h of the same group. Group A = control group; Group B = Dlk1 group; Group C = rhNF-κB group.

The cell growth curves shows that Dlk1 increased AECII proliferation while rhNF-κB decreased their proliferation (Figure 3). (Table 2) Results detected by MTT colorimetry showed that cell proliferation ability in Dlk1 group increased significantly than control group (*P* < 0.01), while in rhNF-κB group significantly lower than control group (*P* < 0.05).

**Table 2:** The proliferation rate of cells among every time spots in group A,B,C (value A).

**Cell Proliferation Rate (value A)**						
**Groups**	**24 h**	**48 h**	**72 h**	**96 h**	***F***	***P***
Group A	0.241 ± 0.03	0.462 ± 0.08^#^	0.535 ± 0.04	0.540 ± 0.02		
Group B	0.233 ± 0.02	0.699 ± 0.05^*^^#^	0.912 ± 0.08^*^^#^	0.726 ± 0.05^*^	37.16	0.02
Group C	0.267 ± 0.06	0.398 ± 0.03^*^^#^	0.402 ± 0.07^*^	0.380 ± 0.11^*^		

# *Compared with preceding time spots of the same group P < 0.05*,

* *compared with the same time spots of the normal group P < 0.05*.

**Figure 4:**
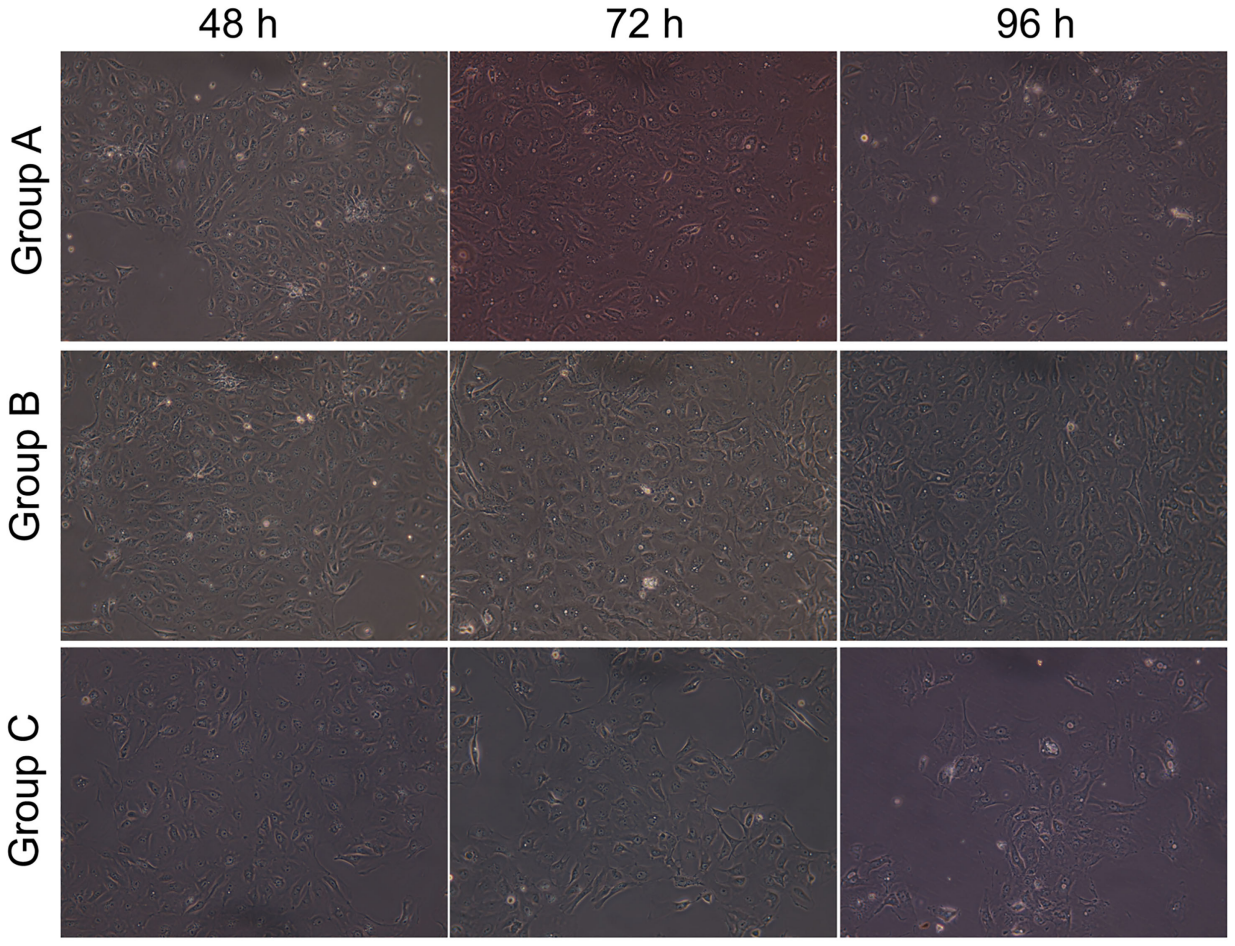
Effect of Dlk1 and rhNF-κB on the morphology of primary cultured rat type II alveolar epithelial cells (AECII). AECIIs were divided into three groups: (1) the control group, cultured with DMEM, and 12% FBS; (2) the Dlk1 group: cells were cultured with Dlk1 (Abnova, Taipei, Taiwan) at a final concentration of 100 ng/mL; and (3) the rhNF-κB group: cells were cultured with rhNF-κB (Promega, Madison, WI, USA) at a final concentration of 1 gsu/ml. Cells were incubated for 48, 72, and 96 h. Cell morphology was observed under inversion microscope (magnification × 200). Group A = control group; Group B = Dlk1 group; Group C = rhNF-κB group.

All cells looked like islands at 24 h; the nuclei and nucleoli were clear, and many particles could be observed in the cytoplasm. Figure 4 shows that at 48 h, in the control group, the cell volume was increased, their number was increased, visible dual-core cells were visible, island-like cells grew to the peripheral region, and the cells had a paving stone appearance (Figure 4-Group A). At 72 h, AECIIs were larger, but their number had no change (Figure 4-Group A). At 96 h, the cell stretch was more pronounced, they became wide, flat, and thin, and a few cells showed pseudopodia or cavity change (Figure 4-Group A). In the Dlk1 group, at 48 h, cell proliferated greatly, more dual-core cells appeared, and cell volume was increased (Figure 4-Group B). At 72 h, the cell number was still increasing, a few cells had a paving stone appearance, and their size was bigger (Figure 4-Group B). At 96 h, the cells started to transdifferentiate (Figure 4-Group B). In the rhNF-κB group, at 48 h, the cells were larger than control cells (Figure 4-Group C). At 72 h, the cell became to stretch, and a few cells had vacuoles (Figure 4-Group C). At 96 h, cell form was highly irregular and the number of suspended cells was increased (Figure 4-Group C).

Taken together, these results suggest that Dlk1 facilitated AECII proliferation, while rhNF-κB reduced AECII proliferation.

### Dlk1 Inhibited AECII Transdifferentiation, While rhNF-κB Promoted This Process

The expression of SP-C (green) and AQP5 (red) fluorescence was observed by laser scanning confocal microscope (LSCM; Figure 1C). In the control group, SP-C expression in the cytoplasm was visible at 48 h, while AQP5 expression was low. At 72 h, the expression of SP-C in the cytoplasm was lower, accompanied by higher AQP5 expression, suggesting that the cells were in an intermediate state of transdifferentiation from AECII to AECI. At 96 h, AQP5 expression was higher, while SP-C was mostly negative, showing that most of the cells had transdifferentiated to AECI (Figure 1C-Group A).

In the Dlk1 group, strong expression of SP-C could be seen at 48 h, while AQP5 expression was negative. After 72 h, the expression of SP-C was still strong, while AQP5 expression was mostly negative, suggesting that the cells did not begin to transdifferentiate and kept the AECII phenotype. At 96 h, cells with double expression of SP-C and AQP5 could be seen, indicating that the cells had begun to transdifferentiate and were in an intermediate state of transdifferentiation (Figure 1C-Group B).

In the rhNF-κB group, expression of SP-C could be seen in the cytoplasm at 48 h, and expression of AQP5 was weak, suggesting that the cells had already began to transdifferentiate. At 72 h, the expression of AQP5 was increased, while SP-C was decreased. It showed that most cells had transdifferentiated to AECI. At 96 h, SP-C had disappeared, AQP5 had also weakened or even disappeared. These results suggest that part of the transdifferentiated AECIs were close to apoptosis(Figure 1C-Group C).

**Figure 5:**
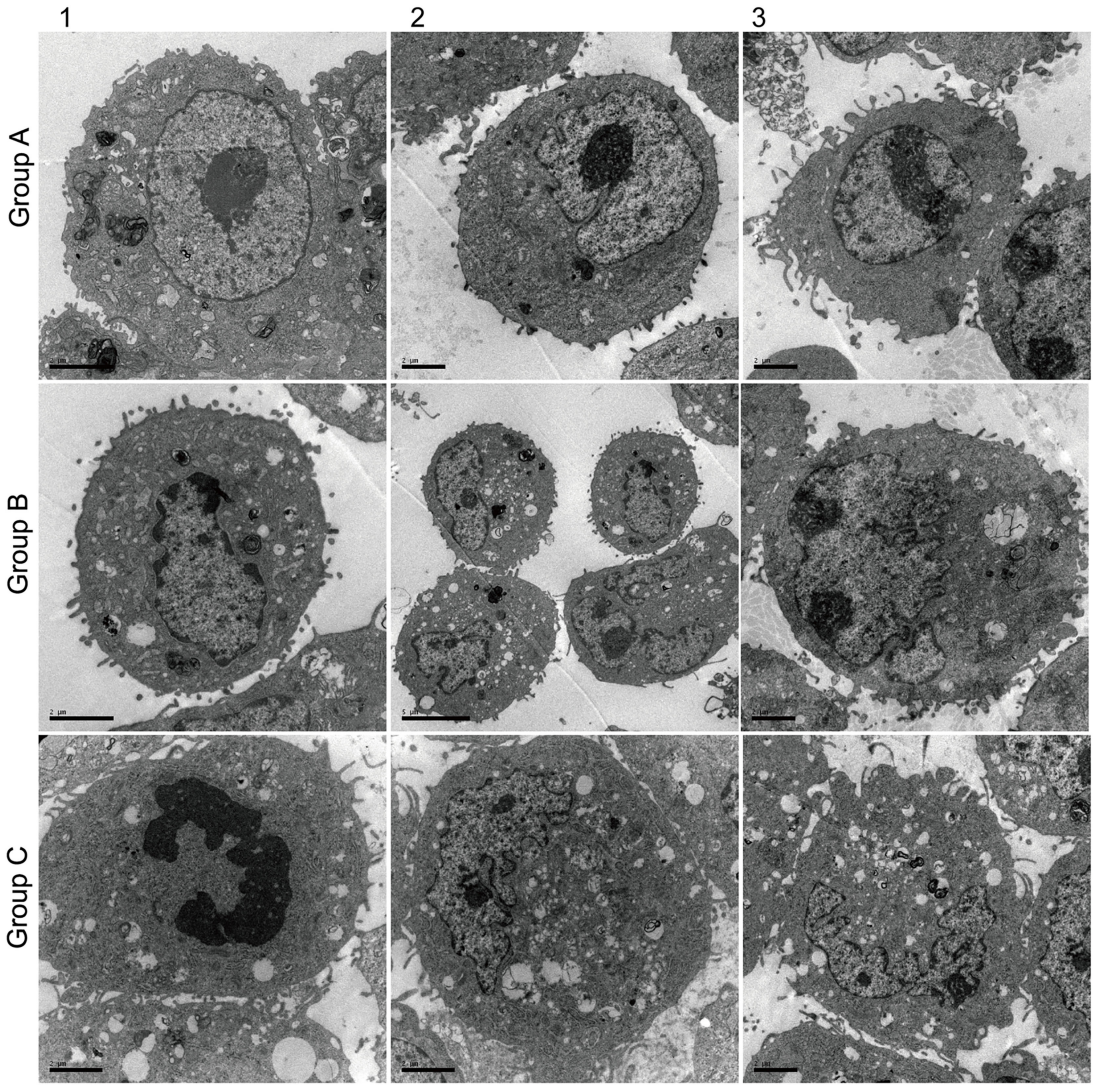
Effect of Dlk1 and rhNF-κB on the ultramicrostructure of primary cultured rat type II alveolar epithelial cells (AECII). AECIIs were divided into three groups: (1) the control group, cultured with DMEM and 12% FBS; (2) the Dlk1 group: cells were cultured with Dlk1 (Abnova, Taipei, Taiwan) at a final concentration of 100 ng/mL; and (3) the rhNF-κB group: cells were cultured with rhNF-κB (Promega, Madison, WI, USA) at a final concentration of 1 gsu/ml. Cells were incubated for 2 h. Ultramicrostructure was determined with a CM10 transmission electron microscope (Philips, Best, The Netherlands) (×12,000). Group A = control group; Group B = Dlk1 group; Group C = rhNF-κB group.

Ultrastructure observation (Figure 5) showed that AECII had layer bodies in the cytoplasm and microvilli on cell surface (Figure 5-Group A). Transdifferentiating AECII had layer bodies in the cytoplasm but no microvilli on cell surface (Figure 5-Group A). Transdifferentiated AECII had microvilli on cell surface, but no layer bodies in the cytoplasm (Figure 5-Group A). AECI showed vacuolar degeneration (Figure 5-Group C).

## Discussion

Transdifferentiation of AECIIs into AECIs is involved in NRDS. Different ligands of the Notch pathway could have different effects on AECII transdifferentiation. Therefore, the present study aimed to investigate the effects of Dlk1 and Jagged1 on the proliferation and transdifferentiation of AECII. The results suggest that Dlk1 promoted proliferation of AECIIs and inhibited cell transdifferentiation, while Jagged1 treatment inhibited proliferation of AECIIs and promoted transdifferentiation to AECIs. These results provide some clue for the eventual management of NDRS.

AECIIs cover about 5% of the alveolar surface, account for 60% of the alveolar epithelial cells and for 15% of the peripheral lung tissue cells (2). AECIIs can not only self-renew and proliferate to form new AECIIs, but they also can undergo transdifferentiation to AECIs under the conditions of alveolar epithelial injury to repair the alveolar epithelium (8). Nevertheless, the most important role of AECIIs is the synthesis and secretion of active substances on lung surface that are essential to the functions of the lung, such as maintaining normal lung water transport, proliferation after injury of alveolar epithelial cells, maintaining alveolar wall integrity, and ensuring normal gas exchange (14). In accordance with these characteristics, the present study showed that transdifferentiation of AECIIs led to decreased expression of SP-C and AQP5, as well as marked changed in the cellular ultrastructure.

Notch is highly conserved in evolution and consists of three parts: receptor, ligand, and DNA binding protein. The activation of Notch signaling pathway relies on the combination of its receptor and ligand (15, 16). In mammals, the receptors include Notch1-4 and the ligands include DLLl-4 and Jagged l. Studies showed that Notch signaling can control cell fate through signal induction as well as lateral differentiation. The substantive characteristics of interaction between cells mediated by Notch lie in the different expression of receptors and ligands between adjacent cells (17, 18). Researches on Drosophila suggest that normal development is unusually sensitive to the expression of Notch and Delta genes. In addition, feedback mechanism between receptor and ligand of the Notch signaling system amplify the differences of expression of Notch and its ligand in the process of development to determine the different differentiation directions of the cells. Notch1/Hes1 signal regulates the expression of C/EBPα and affects the proliferation and differentiation of AECII after exposure to hyperoxia (19).

Dlk1 is a transmembrane protein belonging to the family of notch/delta/serrat (6–8). Dlk1 is a non-canonical ligand of the Notch pathway (6–8). Activation of Notchl reduces the expression of the target gene Hes1 (20). Dlk1 plays an important role in the development and differentiation of various tissues and cells (21). Dlk1-Mediated Temporal Regulation of Notch Signaling Is Required for Differentiation of Alveolar Type II to Type I Cells during Repair (22). In the present study, Dlk1 was added to the cell culture system. The results showed that compared with the control group, the expression of Notch1 was increased and Jagged1 expression was low or absent. The number of AECIIs increased, transdifferentiation was slowed down, and the cells showed good phenotype maintenance. Those results are consistent with a study by Kobayashi et al. (19) that showed that DLKl is mainly to maintain the proliferation state of undifferentiated cells in tissues.

Jagged1 is a canonical ligand of Notch, and several authors confirmed the expression of Jagged1 and Notch1-4 in the lungs of mice (23). In studies of lymphocyte differentiation, Li et al. (24) showed that Jagged1-2 and Deltal3-4 were expressed differently in the bone marrow and thymus. Different ligands play different roles in lymphoid cell differentiation of the central immune system (24). In the present study, after adding rhNF-κB in the culture system to promote the expression of Jagged1, AECII transdifferentiation was accelerated and their proliferation was inhibited. It suggested that the function of Jagged1 on Notch combination was opposite to the function of Dlk1.

Hes1 is one of the target of Notch signaling, and the expression levels of Hes1 can represent the signal intensity of Notch (19). The mechanism of Hed1 is to recruit transcription co-inhibitors like Groucho/TLE, then hindering the gene expression of differentiation effect, and inhibiting the transcription of the cyclin-dependent kinase inhibitor (CKI) p27Kip1 (22). In the present study, when the expression of Hes1 was increased, cell proliferation of AECIIs was inhibited. When cell transdifferentiation was activated, Hes1 expression was reduced which is contrary to the previous studies presented above. It is possible that factors like Hes1 have different activating or inhibiting actions to various effectors in different tissues. Hes1 is an inhibitor of itself and the automatic regulation function keeps its expression unstable (25).

Of course, the present study is not without limitations. The results were obtained *in vitro* experiments by using an animal cell line, which are required to examine Notch regulation in complex human systems. In addition, only a small panel of factors and regulators were examined and additional studies are necessary to understand the regulation of Notch in AECII and to achieve some management approaches for NDRS.

## Conclusions

In conclusion, these results suggest that Dlk1 promoted proliferation of AECIIs and inhibited cell transdifferentiation, while Jagged1 treatment inhibited proliferation of AECIIs and promoted transdifferentiation to AECIs. These results provide an avenue for further exploration into cellular factors that govern the presentation of NRDS and ultimately possibly some future novel management strategies.

## Data Availability Statement

Datasets are available on request: The raw data supporting the conclusions of this article will be made available by the authors, without undue reservation, to any qualified researcher.

## Ethics Statement

The experiments were approved by the Binzhou Medical University Animal Ethics Committee.

## Author Contributions

XL and XZ: conception and design. XL and CW: administrative support. RG, GZ, JM, and XL: provision of study materials or patients. XZ and GZ: collection and assembly of data. XZ, GZ, XL, and CW: data analysis and interpretation. All authors: manuscript writing and final approval of manuscript.

## Conflict of Interest

The authors declare that the research was conducted in the absence of any commercial or financial relationships that could be construed as a potential conflict of interest.
